# ASO Author Reflections: Optimizing the Dose of Floxuridine for the Adjuvant Treatment of Resected Colorectal Cancer Liver Metastases

**DOI:** 10.1245/s10434-025-17994-3

**Published:** 2025-08-06

**Authors:** Kevin P. Labadie, Laleh G. Melstrom

**Affiliations:** Department of Surgery, City of Hope National Medical Center, Duarte, CA USA

## Past

Hepatic arterial infusion (HAI) of floxuridine prolongs survival in select patients after complete resection of colorectal cancer liver metastases (CRLM).^1^ Floxuridine is the drug of choice for HAI owing to high first-pass hepatic metabolism, enabling administration of high doses with limited systemic toxicity. Floxuridine is most commonly administered through a subcutaneous pump into the hepatic arterial system via a surgically implanted catheter in the gastroduodenal artery. Dosing floxuridine is based on the patient’s weight and the pump flow rate, and a standard dose of 0.12 mg/kg/d has been adopted in the adjuvant setting.^2^

Owing to its arterial administration, hepatic and biliary injury are the most common adverse events during treatment with floxuridine. The damage is often self-limited with dose reduction; however, in certain cases, it may cause biliary sclerosis and stricture, which can alter the patient’s future treatment.^3^ To mitigate toxicity, several protective measures have been adopted, including coadministration of dexamethasone, avoiding concurrent antivascular endothelial growth factor (VEGF) therapy, prophylactic ursodiol, and diligent dose reductions on the basis of liver function tests.^4^

Dose reductions are common during floxuridine treatment and can occur in up to 50–90% of patients. The characteristics, risk factors, and oncologic impact of these dose reductions are poorly defined in the adjuvant treatment of CRLM. With increasing utilization of floxuridine, a better understanding of these factors may optimize treatment. The objective of our study was to examine the characteristics and risk factors associated with dose modifications and their impact on oncological outcomes during the adjuvant treatment of resected CRLM.

## Present

Our single-institution, retrospective study examined the dosing characteristics and oncologic outcomes of 71 high-risk patients who received adjuvant floxuridine therapy after complete resection of CRLM over the last 10 years.^5^ An important initial observation was the transition from the standard dose (0.12 mg/kg/d) to a reduced starting dose (0.08 mg/kg/d) around 2021 owing to frequent toxicity and dose reductions associated with the standard dosing. Indeed, 82% of patients require at least one dose reduction when treated with the standard dose, compared with just 53% of patients started on the reduced dose. The magnitude of dose reduction was higher with the standard dose, with most patients receiving less than 80% of the intended dose intensity. The dose reductions were owing to more severe liver function abnormalities, in which four patients (10%) developed biliary sclerosis with standard dosing. Of the examined risk factors, female sex was associated with more frequent dose reductions, but no other individual or cancer characteristics were associated. This may be owing to the relatively small sample size of our cohort. To our knowledge, these data are the first to report differences in treatment tolerability between floxuridine doses in the adjuvant treatment setting.

We then sought to examine the impact of the reduced floxuridine dose on survival. Although patients started on reduced dose had shorter median follow-up, we observed preserved short-term hepatic-recurrence-free and overall survival compared with standard dosing. Longer follow-up is needed to confirm these findings. A selection bias may have influenced our findings; however, we believe the bias is minimal owing to similarity of patient cohorts and the starting dose was uniformly reduced to 0.08 mg/kg/d for all patients in the latter part of the treatment.

On the basis of these observations, our treatment team advocates the reduced dose of 0.08 mg/kg/d as the new standard for the adjuvant treatment of resected CRLM.

## Future

The use of hepatic arterial infusion is increasing worldwide for the management of CRLM. In many instances, it is utilized in earlier phases of treatment such as in the neoadjuvant and primary adjuvant setting. Therefore, mitigating long-term biliary toxicity is critical to ensure treatment longevity and the availability of salvage therapies. This is even more important as the incidence of CRLM is increasing in younger patients, and there is a greater need for prolonged tolerable and effective therapies. Indeed, some patients can have their HAI pump indefinitely for repeat use in the salvage setting. Further research is needed to understand the exact mechanisms of biliary toxicity to develop better protective treatments. Dose reduction may be a good initial step but is insufficient, as toxicity persists even at lower doses. As more centers adopt HAI therapy, registries and larger cohorts should be developed to collaborate and innovate to enhance the care of our patients.
